# High-speed lateral stability and trajectory tracking performance for a tractor-semitrailer with active trailer steering

**DOI:** 10.1371/journal.pone.0277358

**Published:** 2022-11-14

**Authors:** Yu Hou, Xiaomei Xu

**Affiliations:** College of Automobile and Traffic Engineering, Nanjing Forestry University, Nanjing, China; Beijing Institute of Technology, CHINA

## Abstract

An active trailer steering (ATS) controller is investigated to improve the lateral stability and trajectory tracking performance of the tractor-semitrailer. First of all, a linear yaw-roll dynamic model of the tractor-semitrailer with steerable trailer wheels is established, and the model accuracy is verified. Then a linear quadratic regulator (LQR) for actively steering the trailer’s wheels is designed. For the LQR controller, the lateral acceleration and the sideslip angle at the center of gravity (CG) of the trailer are taken as the optimization objectives, and the steering angle of the wheel on the middle axle of the trailer is set as the control input. Finally, the effectiveness of the designed controller is tested based on the co-simulation platform under the single lane-change (SLC) maneuver at the speed of 100 km/h and the double lane-change (DLC) maneuver at the speed of 80 km/h and 88km/h. Research results show that under the high-speed SLC maneuver, the designed LQR controller can significantly improves the lateral stability and trajectory tracking performance of the trailer, and cannot affect apparently the trajectory and dynamic responses of the tractor. Under the high-speed DLC maneuver, the designed controller can still make the tractor-semitrailer reach a new steady state in a short time, and improve the vehicle lateral stability and the trajectory tracking performance of the trailer at the same time.

## 1 Introduction

Tractor-semitrailers have become one of the most essential means in highway transportation because of their large cargo capacity and good transportation economy. However, due to their sizeable dead weight and volume, the high center of gravity (CG), and the mutual interference of force and motion between the tractor and the trailer, the tractor-semitrailers are prone to lateral instability and serious traffic safety accidents [1–3]. Given this, scholars at home and abroad have carried out a large number of studies on the improvement of high-speed lateral stability of the tractor-semitrailers and the trajectory tracking performance of the trailers.

Existing methods for improving vehicles’ lateral stability or the trajectory tracking performance mainly include the active suspension method [4–6], the compliance steering or passive steering of the rear axles or wheels [7–9], the differential braking method [10, 11], the active steering of the rear axles or wheels [12–14], and the parameter and state estimation for vehicles [15–17]. The active suspension method usually consumes a lot of energy, and the response speed is relatively slow, which is not practical for articulated heavy vehicles (AHVs). The compliance steering or passive steering of the rear axles or wheels works well in steady-state circles at low speeds, while during transient low-speed maneuvers and at high speeds it maybe generate inappropriate steer angles. Simulations and tests indicate that the differential braking method can improve maneuverability and enhance the lateral stability of tractor-semitrailers under emergency maneuvers at high lateral accelerations [18, 19]. However, activating a trailer differential braking system makes a continuous and fine-tuned control intervention impossible, leading to undesired speed reduction and excessive wear of the braking lining and tires [20]. An ATS system can be effectively complementary to the trailer differential braking system; ATS systems can continuously operate under normal and high-speed evasive maneuvers with non-aggressive intervention to suppress exaggerated lateral motions of rearward trailers [21].

ATS systems have three kinds of controllers for the tractor-semitrailers, namely, improving the path-following performance, improving the high-speed lateral stability, and a combination of both. The path-following performance is often measured by the path-following off-tracking (PFOT), which refers to a maximum radial offset between the path of the front-axle center of a truck or tractor and the path of the rear-axle center of the rearmost trailer under a given test manoeuver [22]. The high-speed lateral stability is usually measured by the rearward amplification (RWA), which is defined as a ratio of the peak lateral acceleration at the CG of the rearmost trailer to that of the tractor under an obstacle avoidance lane-change maneuver [23]. Kharrazi et al. proposed a steering-based controller to steer the axles of the towed units of longer combination vehicles. Research results showed that the controller reduced the yaw rate RWA and off-tracking considerably without diminishing the manoeuvrability [24]. Then, the effectiveness and robustness of the controller were verified in various driving conditions at high speeds and presence of uncertainties in vehicle parameters [25]. Jujnovich et al. focused on achieving accurate path following for tractor and trailer, for all paths and all normal vehicle speeds [26]. They studied an active steering control strategy for AHVs and blended the low and high speed controllers together using a speed-dependent gain. Kim et al. proposed an active steering controller based on LQR to make the tractor and trailer follow the desired yaw angle and sideslip angle by actively steering the tractor’s rear axle and the trailer’s axles [27]. Kural et al. employed a single controller to solve the combined problem of low-speed maneuverability and high-speed stability of high-capacity vehicles. Simulation results showed a substantial reduction of the swept path width and tail swing for low speeds and the RWA for high speeds [28]. Ni et al. designed a robust ATS controller for multi-trailer AHVs using a linear matrix inequality-based LQR method. The numerical and hardware-in-the-loop real-time simulation demonstrated that the robust ATS controller was effective for increasing the safety of MTAHVs, and robust under varied operating conditions [29]. Zhu et al. studied the robustness of different controllers for ASSs of AHVs, and evaluated the controllers using numerical simulation in terms of the trade-off between maneuverability and lateral stability at high speeds [30]. Marumo et al. used a vector follower controller to compensate the direction difference angles between the velocity vector at the front articulation point and the trailer using active steering of the trailer wheels, and applied a lane-keeping-assistance system to the tractor to suppress the lateral deviation of the tractor and the steering effort of the driver [31].

Generally, many controllers have been developed for the tractor-semitrailers, and more research focused on the low-speed path-following performance and high-speed stability of the tractor-semitrailers. In fact, the high-speed trajectory tracking performance of trailers also has an essential impact on the safe driving of the tractor-semitrailers. Roebuck et al. designed a high-speed path-following controller for long combination vehicles, and implemented it on a test vehicle [32]. The results showed reduction in lateral tracking error, lateral acceleration RWA and yaw rate RWA. Kati et al. proposed a gain-scheduled controller for AHVs by actively steering the selected towed vehicle units. Simulation results confirmed a significant reduction in yaw rate RWA, lateral acceleration RWA, and high-speed transient off-tracking [33]. It is worth mentioning that, most of the above studies are based on the plane model of the tractor-semitrailer, without considering the roll motion of the vehicle.

The motivations and contributions in this study focus on two aspects. The first purpose is to design a control system aiming at improving the lateral stability of the tractor-semitrailer and the trajectory tracking performance of the trailer under high-speed lane-change maneuvers. The second purpose is to reveal the effectiveness of the controller designed based on the linear yaw-roll model for controlling vehicle stability and the trajectory tracking performance of the trailer under the SLC and DLC maneuvers at high speeds. Therefore, in this study the linear yaw-roll model of the tractor-semitrailer is adopted for the design of stability controller, the lateral acceleration and sideslip angle at the trailer’s CG are taken as the optimization objectives to formulate a general cost function. Based on the co-simulation platform the effectiveness of the designed controller is examined under the high-speed SLC and DLC maneuvers.

The rest of this article is organized as follows. In section 2, the linear yaw-roll model of the tractor-semitrailer is introduced. In section 3, the controller for the ATS based on the LQR is designed. In section 4, numerical experiments of the tractor-semitrailer are performed under the SLC and DLC maneuvers at high speeds. Finally, concluding remarks are drawn in section 5.

### 2 Linear yaw-roll model of the tractor-semitrailer

**Fig 1:**
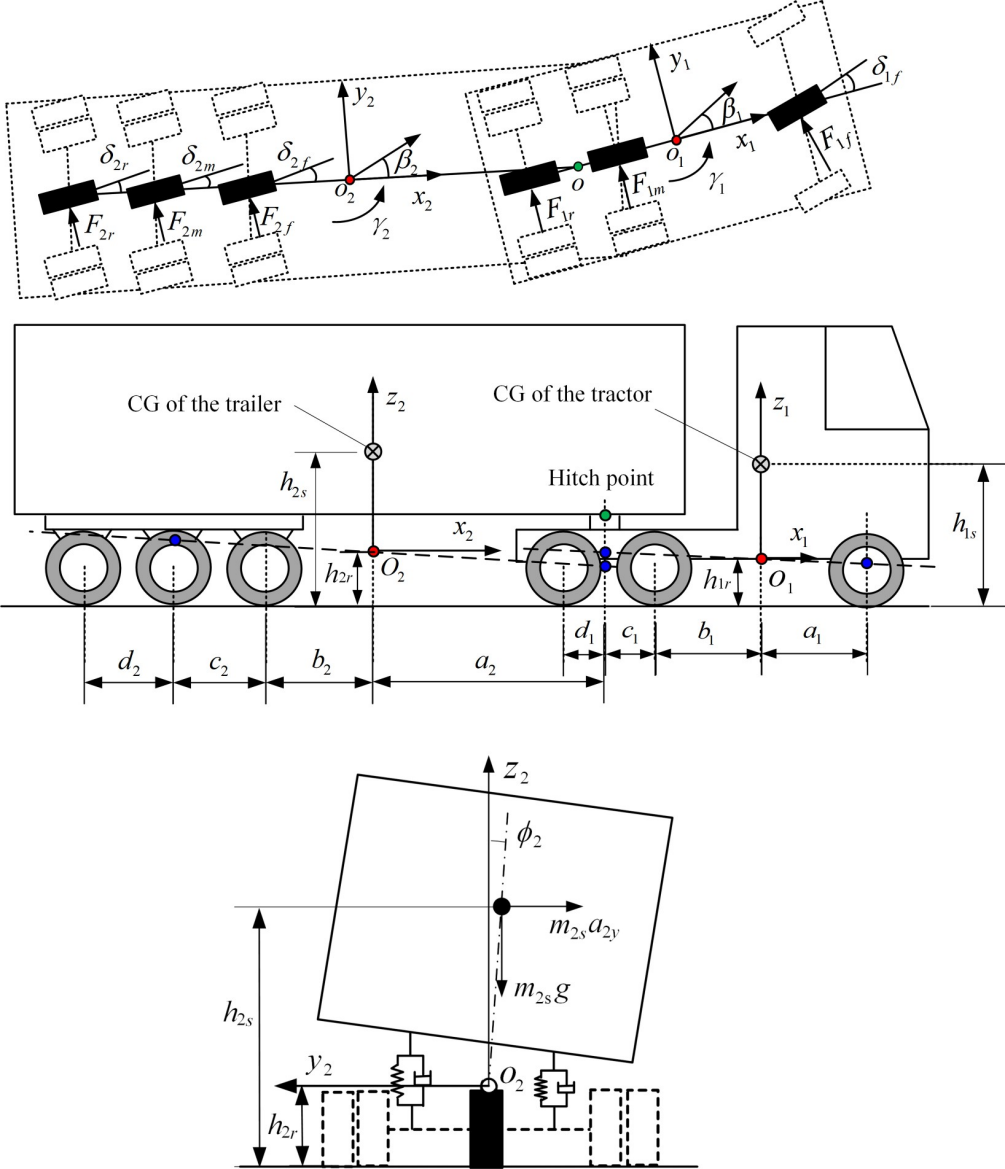
Schematic representation of the tractor-semitrailer model with the trailer steering system. (*a*) Top view. (*b*) Side view. (*c*) Rear view.

To study the dynamic characteristics of the tractor-semitrailer and design a stability controller, a linear yaw-roll model is generated to represent the tractor-semitrailer. Fig 1 shows the schematic representation of the tractor-semitrailer model, in which each axle is represented by a single wheel. In the figure, *x*_1_ –*y*_1_ *–z*_1_ and *x*_2_ –*y*_2_ *–z*_2_ are the body-fixed coordinate systems for the tractor and trailer, respectively; *δ*_1*f*_ is the front wheel steering angle of the tractor; *δ*_2*f*_, *δ*_2*m*_, and *δ*_2*r*_ represent the steering angles of the front, middle and rear axles of the trailer, respectively; *β*_1_ and *β*_2_ represent the sideslip angles at the tractor’s CG and trailer’s CG, respectively; *γ*_1_ and *γ*_2_ denote the yaw rates of the tractor and trailer, respectively; *ϕ*_1_ and *ϕ*_2_ are the roll angles of the sprung mass of the tractor and trailer, respectively. Description of the other parameters of the tractor-semitrailer is given in S1 Appendix.

To derive the linear dynamic model of the tractor-semitrailer, the assumptions have been made as follows: (1) the forward speeds of the tractor and the semitrailer are identical, and at a constant speed under the simulated test maneuvers; (2) the steering angle *δ*_1*f*_ of the tractor front wheel is small; (3) the pitch and bounce motions, braking forces, and aerodynamic forces are ignored; (4) the articulation angle *θ* between the tractor and the semitrailer is small; (5) the roll stiffness and damping coefficients of the suspension systems for the tractor-semitrailer are constant in the range of roll motions involved. Based on the coordinate systems *x*_1_ –*y*_1_ *–z*_1_ and *x*_2_ –*y*_2_ *–z*_2_, the governing equations for motions of the tractor and trailer can be generated in Eqs (1) and (2).

$$\{\begin{array}{l} m_{1}v_{x1}({\dot{\beta }}_{1}+\gamma_{1})-m_{1s}h_{1sr}{\ddot{\phi }}_{1}=F_{1f}+F_{1m}+F_{1r}-F_{1oy}\\ I_{1zz}{\dot{\gamma }}_{1}-I_{1sxz}{\ddot{\phi }}_{1}=F_{1f}a_{1}-F_{1m}b_{1}-F_{1r}(b_{1}+c_{1}+d_{1})+F_{1oy}(b_{1}+c_{1})\\ \left[I_{1sxx}+m_{1s}h_{1sr}{}^{2}]{\ddot{\phi }}_{1}-I_{1sxz}{\dot{\gamma }}_{1}=m_{1s}h_{1sr}[v_{x1}({\dot{\beta }}_{1}+\gamma_{1})-h_{1sr}{\ddot{\phi }}_{1}]+m_{1s}gh_{1sr}\phi_{1}\right)\\ \;-K_{1}^{*}\phi_{1}-C_{1}^{*}{\dot{\phi }}_{1}+K_{12}(\phi_{2}-\phi_{1})+F_{1oy}h_{1cr} \end{array}$$ (1)

$$\{\begin{array}{l} m_{2}v_{x2}({\dot{\beta }}_{2}+\gamma_{2})-m_{2s}h_{2sr}{\ddot{\phi }}_{2}=F_{2f}+F_{2m}+F_{2r}+F_{2oy}\\ I_{2zz}{\dot{\gamma }}_{2}-I_{2sxz}{\ddot{\phi }}_{2}=-F_{2f}b_{2}-F_{2m}(b_{2}+c_{2})-F_{2r}(b_{2}+c_{2}+d_{2})+F_{2oy}a_{2}\\ \left[I_{2sxx}+m_{2s}h_{2sr}{}^{2}]{\ddot{\phi }}_{2}-I_{2sxz}{\dot{\gamma }}_{2}=m_{2s}h_{2sr}[v_{x2}({\dot{\beta }}_{2}+\gamma_{2})-h_{2sr}{\ddot{\phi }}_{2}]+m_{2s}gh_{2sr}\phi_{2}\right)\\ \;-K_{2}^{*}\phi_{2}-C_{2}^{*}{\dot{\phi }}_{2}-K_{12}(\phi_{2}-\phi_{1})-F_{2oy}h_{2cr} \end{array}$$ (2)

where *F*_1*oy*_ and *F*_2*oy*_ are the lateral reaction forces of the hitch acting on the tractor and the semitrailer, respectively; *h*_1*cr*_ and *h*_2*cr*_ are the distances between the hitch point and the roll center of the sprung mass for the tractor and semitrailer, respectively; *h*_1*sr*_ and *h*_2*sr*_ are the distances between the CG and the roll center of the sprung mass for the tractor and semitrailer, respectively; *v*_*x*1_ and *v*_*x*2_ are longitudinal velocities of the tractor and semitrailer, respectively.

The mechanical constraint equations between the tractor and the semitrailer are expressed as

$$\left\{ \begin{array}{l} F_{1ox}=F_{2ox}\cos\theta +F_{2oy}\sin\theta \\ F_{1oy}=F_{2oy}\cos\theta -F_{2ox}\sin\theta \end{array}\right.$$ (3)

where *F*_1*ox*_ and *F*_2*ox*_ are the longitudinal reaction forces of the hitch acting on the tractor and the semitrailer, respectively.

The kinematic constraint equations between the tractor and the semitrailer can be described as

$$\left\{ \begin{array}{l} v_{x1}\cos\theta -[v_{y1}-(b_{1}+c_{1})\gamma_{1}-h_{1cr}{\dot{\phi }}_{1}]\sin\theta =v_{x2}\\ v_{x1}\sin\theta +[v_{y1}-(b_{1}+c_{1})\gamma_{1}-h_{1cr}{\dot{\phi }}_{1}]\cos\theta =v_{y2}+a_{2}\gamma_{2}-h_{2cr}{\dot{\phi }}_{2} \end{array}\right.$$ (4)

where *v*_*y*1_ and *v*_*y*2_ are lateral velocities of the tractor and semitrailer, respectively.

Since the articulation angle *θ* is assumed to be small, sin*θ*≈0 and cos*θ*≈1. According to the definition of sideslip angle, Eq (4) can be further written as

$${\dot{\beta }}_{2}={\dot{\beta }}_{1}-\frac{(b_{1}+c_{1}){\dot{\gamma }}_{1}}{v_{x1}}-\frac{h_{1cr}{\ddot{\phi }}_{1}}{v_{x1}}-\frac{a_{2}{\dot{\gamma }}_{2}}{v_{x2}}+\frac{h_{2cr}{\ddot{\phi }}_{2}}{v_{x2}}+\gamma_{1}-\gamma_{2}$$ (5)

In this study, the linear tire model is used to represent the relationship between the tire lateral force and tire sideslip angle, which can be written as

$$\{\begin{array}{c} F_{1f/m/r}=k_{1f/m/r}\cdot \alpha_{1f/m/r}\\ F_{2f/m/r}=k_{2f/m/r}\cdot \alpha_{2f/m/r} \end{array}$$ (6)

where *F*_1*f/m/r*_ and *F*_2*f/m/r*_ denote the lateral forces subjected by the front, middle and rear axles for the tractor and trailer, respectively; *k*_1*f/m/r*_ and *k*_2*f/m/r*_ denote the tire cornering stiffness of the front, middle and rear axles for the tractor and trailer, respectively. *α*_1*f/m/r*_ and *α*_2*f/m/r*_ represent the tire sideslip angles for the front, middle and rear axles for the tractor and trailer, respectively.

The tire sideslip angles of each axle of the tractor-semitrailer can be described as

$$\{\begin{array}{l} \alpha_{1f}=\beta_{1}+a_{1}\gamma_{1}/v_{x1}-\delta_{1f}\\ \alpha_{1m}=\beta_{1}-b_{1}\gamma_{1}/v_{x1}\\ \alpha_{1r}=\beta_{1}-(b_{1}+c_{1}+d_{1})\gamma_{1}/v_{x1}\\ \alpha_{2f}=\beta_{2}-b_{2}\gamma_{2}/v_{x2}-\delta_{2f}\\ \alpha_{2m}=\beta_{2}-(b_{2}+c_{2})\gamma_{2}/v_{x2}-\delta_{2m}\\ \alpha_{2r}=\beta_{2}-(b_{2}+c_{2}+d_{2})\gamma_{2}/v_{x2}-\delta_{2r} \end{array}$$ (7)

When the tractor-semitrailer runs at high speeds, the steering angles of the wheels on the trailer’s three axles must satisfy a specific relationship. The steering angles of the trailer can be allocated based on the principle of making the sideslip forces on the trailer wheels equal. Since the cornering stiffness of all trailer tires is approximately the same, the relationship among the three steering angles of the trailer wheels can be written as

$$\{\begin{array}{c} \delta_{2f}=c_{2}\gamma_{2}/v_{x2}+\delta_{2m}\\ \delta_{2r}=-d_{2}\gamma_{2}/v_{x2}+\delta_{2m} \end{array}$$ (8)

If the steering angle *δ*_2m_ of the trailer middle axle is set as the control vector, the linear vehicle model can be expressed in the state-space form as Eq (9), in which ***u*** is the control input, ***u*** = [*δ*_2m_].

$$\dot{X}=AX+Bu+B_{0}\delta_{1f}$$ (9)

where ***A***, ***B*** and ***B***_**0**_ are the system, the control, and the disturbance matrices, respectively. These matrices and their non-zero elements are presented in S2 Appendix. The state variable vector ***X*** is defined as

$$X={\left[\beta_{1}\;\gamma_{1}\;\phi_{1}\;{\dot{\phi }}_{1}\;\beta_{2}\;\gamma_{2}\;\phi_{2}\;{\dot{\phi }}_{2}\right]}^{T}$$ (10)

Due to the yaw-roll vehicle model is a simplified description for the tractor-semitrailer, it is necessary to verify the accuracy of the vehicle model. The model accuracy verification is carried out by the TruckSim software under high-speed SLC maneuvers. The verification results show an excellent consistency of the dynamic responses from the yaw-roll model and the TruckSim model. Therefore, the yaw-roll model of the tractor-semitrailer can be adopted for the design of the stability controller in the following section.

## 3 Design of the active steering controller

In this section, a linear quadratic regulator (LQR) is designed to improve the high-speed lateral stability of the tractor-semitrailer. LQR design is an optimization problem that minimizes the performance index under given constraints and obtains the optimal feedback controller by solving the corresponding algebraic Riccati equation. The cost function of LQR problem design in infinite time is written as Eq (11).

$$J=\int_{0}^{\infty }\left(x^{T}Qx+u^{T}Ru\right)dt$$ (11)

where ***x*** is the system state vector, ***u*** is the active control input vector, ***Q*** is the weighting matrix of the system state variables, and ***R*** is the weighting matrix of the system control input.

The optimal control law is given by a feedback state form.

$$u=-Kx=-R^{-1}B^{T}Px$$ (12)

where ***K*** is the optimal gain matrix, ***B*** is the control input matrix, and ***P*** is a symmetric, positive semi-definite symmetric matrix that satisfies the Riccati equation shown in Eq (13).


$$PA+A^{T}P-PBR^{-1}B^{T}P+Q=0$$ (13)

According to the mathematical model of the tractor-semitrailer, the wheel on the tractor’s front axle is steerable, and the ATS angle *δ*_2m_ is to be estimated by the optimal controller based on the LQR technique. For the ATS control design of a tractor-semitrailer, the lateral acceleration of the trailer is usually selected as the index to evaluate the lateral stability of a tractor-semitrailer, but if only the lateral acceleration is taken as the optimization target, the trajectory tracking performance of the trailer to follow the tractor trajectory will deteriorate. Through many simulation experiments, it has been found that when the peak value of the sideslip angle at the trailer’s CG increases sharply, the trajectory tracking performance of the trailer will become poor. If the sideslip angle at the trailer’s CG is constrained, a better trajectory tracking performance can be achieved. Therefore, to improve the lateral stability of the tractor-semitrailer and the trajectory tracking performance of the trailer, the lateral acceleration and sideslip angle at the trailer’s CG are taken as the optimization objectives in this study. The relationship among the trailer’s lateral acceleration, the trailer’s sideslip angle, and the active steering angle is created by forming a general cost function given in Eq (14).

$$J_{1}=\int_{0}^{\infty }\left[W_{1}{\left(\beta_{2}\right)}^{2}+W_{2}{\left(a_{y2}\right)}^{2}+W_{3}{\left(\delta_{2m}\right)}^{2}\right]dt$$ (14)

where *β*_2_ and *a*_*y*2_ represent the sideslip angle and lateral acceleration at the trailer’s CG, respectively; *Wi* (*i* = 1, 2, 3) are the weighting factors affecting the sideslip angle, the lateral acceleration, and the ATS angle, respectively. By adjusting the weightings *W*1, *W*2, and *W*3 for different purposes of the trailer steering control scenario, the weighting factors ***Q*** and ***R*** in Eq (11) are then assigned to minimize the cost function. In this study, *W*_1_, *W*_2_, and *W*_3_ were obtained by repeated simulation tests. The optimal gain vector ***K*** is determined by solving the algebraic Riccati equation to minimize the cost function ***J***_1_. Then, the optimal steering angle of the trailer middle axle can be calculated as

$$\delta_{2m}=-Kx$$ (15)

**Fig 2:**
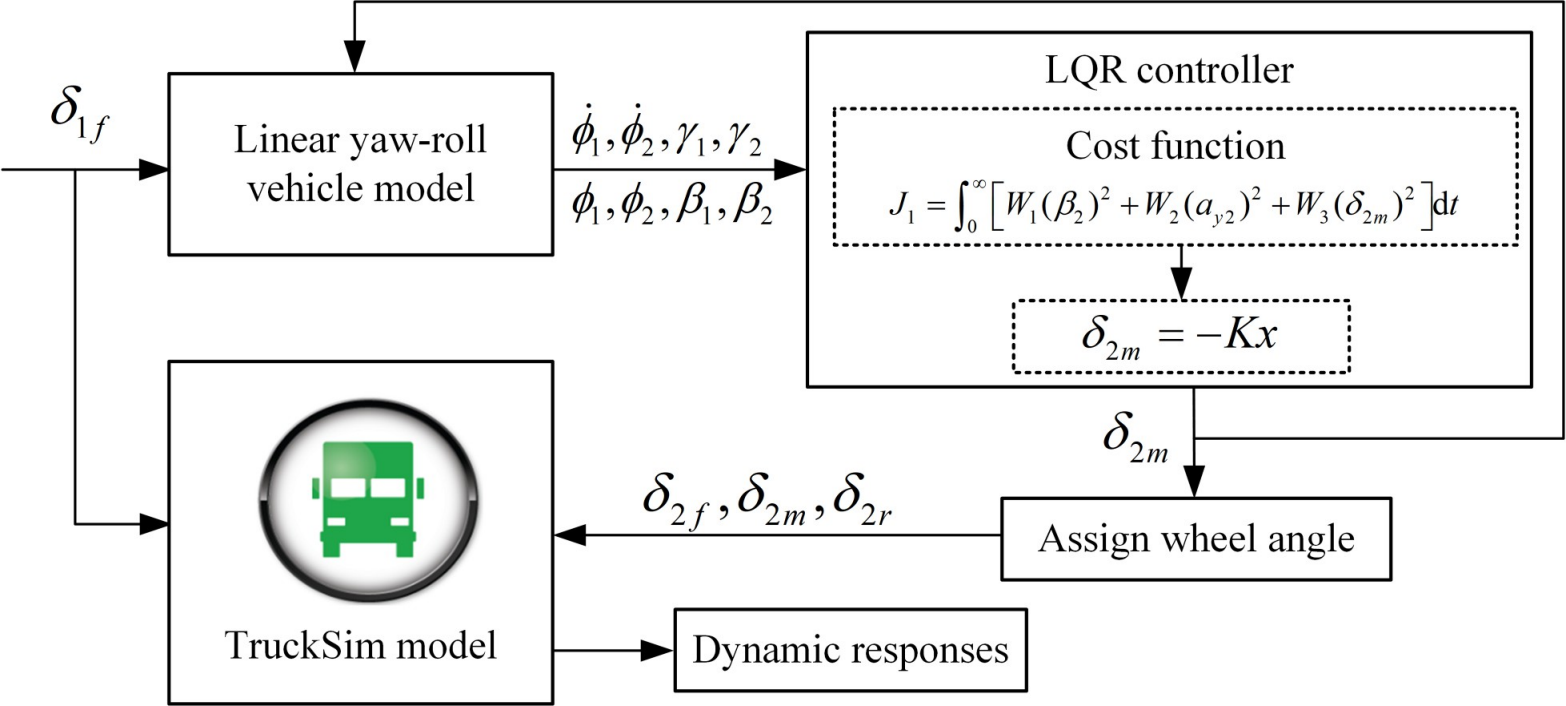
Block diagram of the LQR controller.

To clearly illustrate the implementing process of the control strategy, the block diagram of implementing control strategy is given in Fig 2. The steering angle *δ*_1*f*_ of the tractor front wheel is taken as the input for the linear yaw-roll vehicle model. The LQR controller calculates the optimal trailer steering angle *δ*_2m_. According to Eq (8), the steering angles of the front and rear axles of the semitrailer can be calculated. Then the three steering angles are sent to the TruckSim model to calculate the dynamic responses. At the same time, the steering angle *δ*_2m_ is also sent to the linear yaw-roll vehicle model to calculate the new status information of the tractor-semitrailer and then obtains the new optimal trailer steering angle *δ*_2m_.

## 4 Numerical experiments and discussions

In this section, to evaluate the performance of the proposed LQR controller for the tractor-semitrailer, two typical road test scenarios are applied: a single lane-change (SLC) maneuver and a double lane-change (DLC) maneuver. For above test scenarios, the control process is performed through the Simulink blocks and TruckSim software. The required ATS angle of the trailer is calculated in Simulink and fed to the TruckSim symbolic S-function. The nominal values of the system parameters for the tractor-semitrailer are given in S1 Appendix. To simplify the expression in the following discussion, the tractor-semitrailer without ATS is referred to as the baseline case, and that with ATS is named as the ATS case. The simulation test is carried out on the road with an adhesion coefficient of 0.85.

### 4.1 Single lane-change maneuver

**Fig 3:**
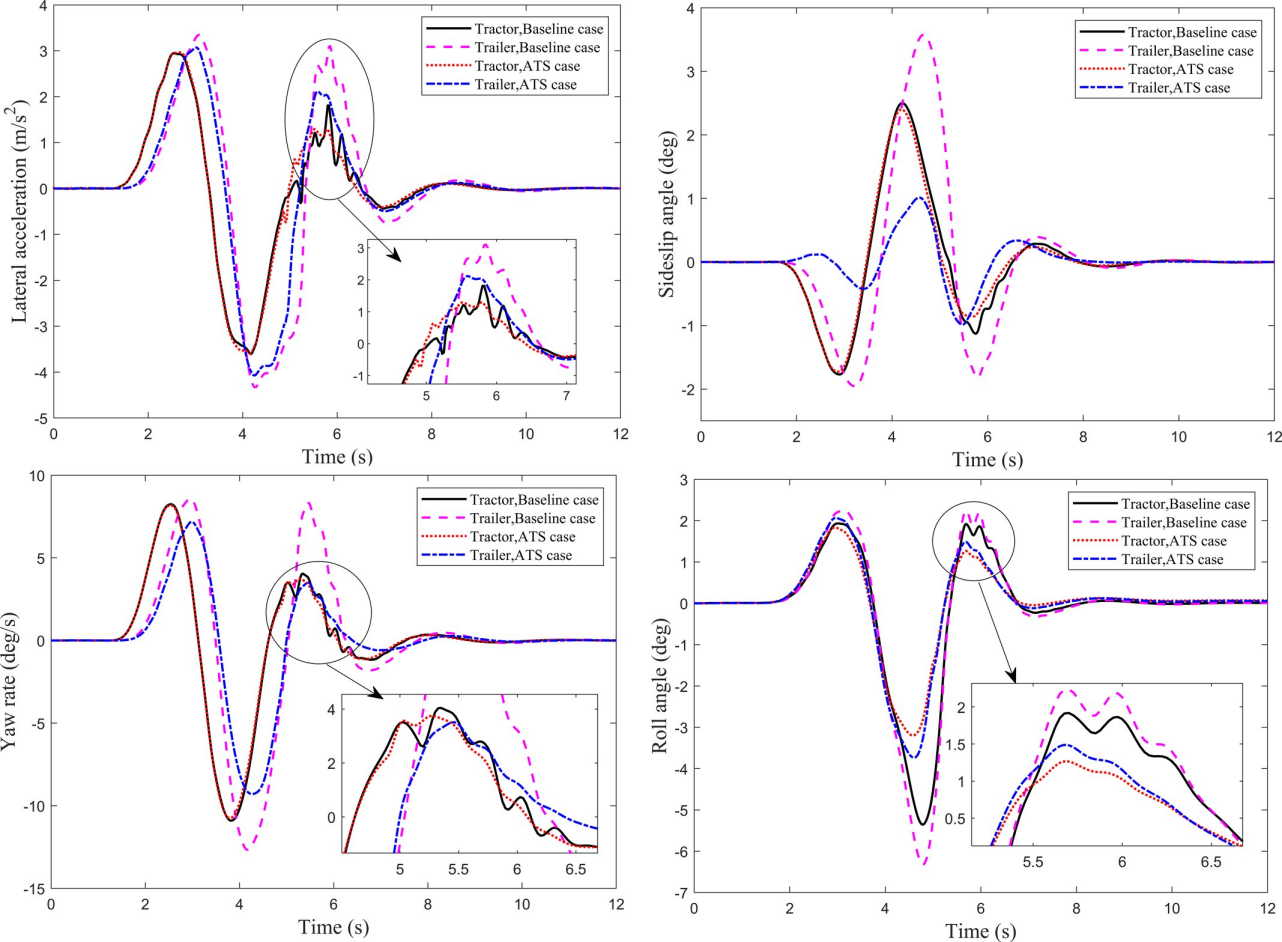
Dynamic responses of the tractor-semitrailer under the SLC maneuver. (*a*) Lateral acceleration. (*b*) Sideslip angle. (*c*) Yaw rate. (*d*) Roll angle.

The Single lane-change (SLC) maneuver of the tractor-semitrailer is performed at 100 km/h. The simulation time step is set to 0.001s, and the simulation time is 12s. The trajectory tracking performance of the trailer and the lateral stability of the tractor-semitrailer with an ATS system are compared with the baseline case. Four dynamic responses shown in Fig 3 are used to describe the lateral stability of the tractor-semitrailer. It can be seen that, except for the roll angle, the dynamic responses of the trailer all lag behind those of the tractor. Fig 3(A) shows the lateral accelerations at CG of the tractor and trailer under the SLC maneuver for the baseline and ATS cases. For the ATS case, the second peak value on the trailer lateral acceleration curve is 2.111 m/s^2^. Compared with the baseline case, 3.102 m/s^2^, the second peak value of the trailer lateral acceleration is reduced by 31.9%. According to the definition of RWA, the calculated RWA is decreased from 1.133 to 1.035. As indicated before, RWA is an important index to evaluate the high-speed lateral stability of the tractor-semitrailer. The lower the RWA, the better the lateral stability. Therefore, the reduction of the RWA indicates that the improved lateral stability of the tractor-semitrailer can be achieved by the designed LQR controller. Moreover, Fig 3(A) also shows that the lateral acceleration curves for the baseline case oscillate obviously during the SLC maneuver, which means that shimmy occurs in the tractor-semitrailer without an ATS system. In other words, the designed LQR controller can suppress the shimmy phenomenon of the tractor-semitrailer in the process of high-speed SLC maneuver.

Fig 3(B)–3(D) show under the SLC maneuver for the baseline case and ATS case, the time history of the sideslip angles at the CG, the time history of the yaw rates and roll angles for the tractor and trailer, respectively. The maximum value of the trailer sideslip angle for the baseline case is 3.57°, and for the ATS case is 1.01°, decreased by 71.7%. The maximum value of the trailer yaw rate for the baseline case is 12.66 deg/s and for the ATS case is 9.29 deg/s, decreased by 26.6%. The ATS system reduces the maximum roll angle of the tractor from 5.36° to 3.20°, decreased by 40.3%, and reduces the maximum roll angle of the trailer from 6.32° to 3.74°, decreased by 40.8%. For the baseline case, the dynamic response curves present apparent oscillation during the high-speed SLC maneuver. The designed LQR controller can effectively suppress the shimmy phenomenon of the tractor-semitrailer in the process of high-speed SLC maneuver.

**Fig 4:**
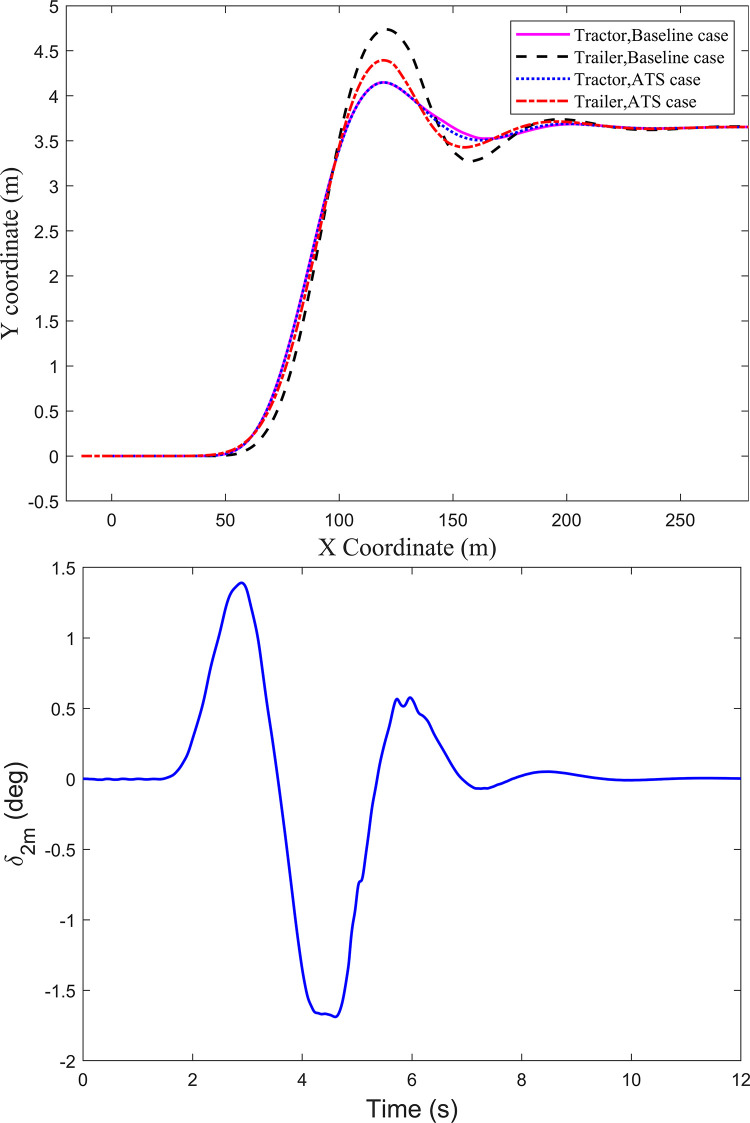
Axle trajectories and the active steering angle under the SLC maneuver. (*a*) Axle trajectories. (*b*) Active steering angle *δ*_2m_.

As shown in Fig 1, the tractor-semitrailer has six axles in total. From the tractor front axle to the rearmost axle of the trailer, they are denoted as A1 to A6. Thus, axle A1 and axle A6 are the front axle of the tractor and the rearmost axle of the trailer, respectively. The trajectories of axle A1 and axle A6 shown in Fig 4(A) are adopted to describe the trajectory tracking performance of the trailer to follow the tractor. It can be seen there is an apparent trajectory deviation between axle A6 and axle A1. For the baseline case, the trajectory of axle A6 deviates from axle A1, leading to the deviation of 0.591 m. However, for the ATS case, the deviation between axles A6 and A1 is 0.247 m, and the improvement ratio is 58.2%. It is obvious that the LQR technique can improve the trajectory tracking performance of the trailer under the high-speed SLC maneuver. To understand the change of the middle-axle active steering angle *δ*_2m_ of the trailer under the SLC maneuver, Fig 4(B) shows its time history curve.

In summary, the designed LQR controller has no noticeable effect on the tractor trajectory and dynamic responses except the roll angle. At the same time, it significantly improves the lateral stability and trajectory tracking performance of the trailer under the high-speed SLC maneuver.

### 4.2 Double lane-change maneuver

**Fig 5:**
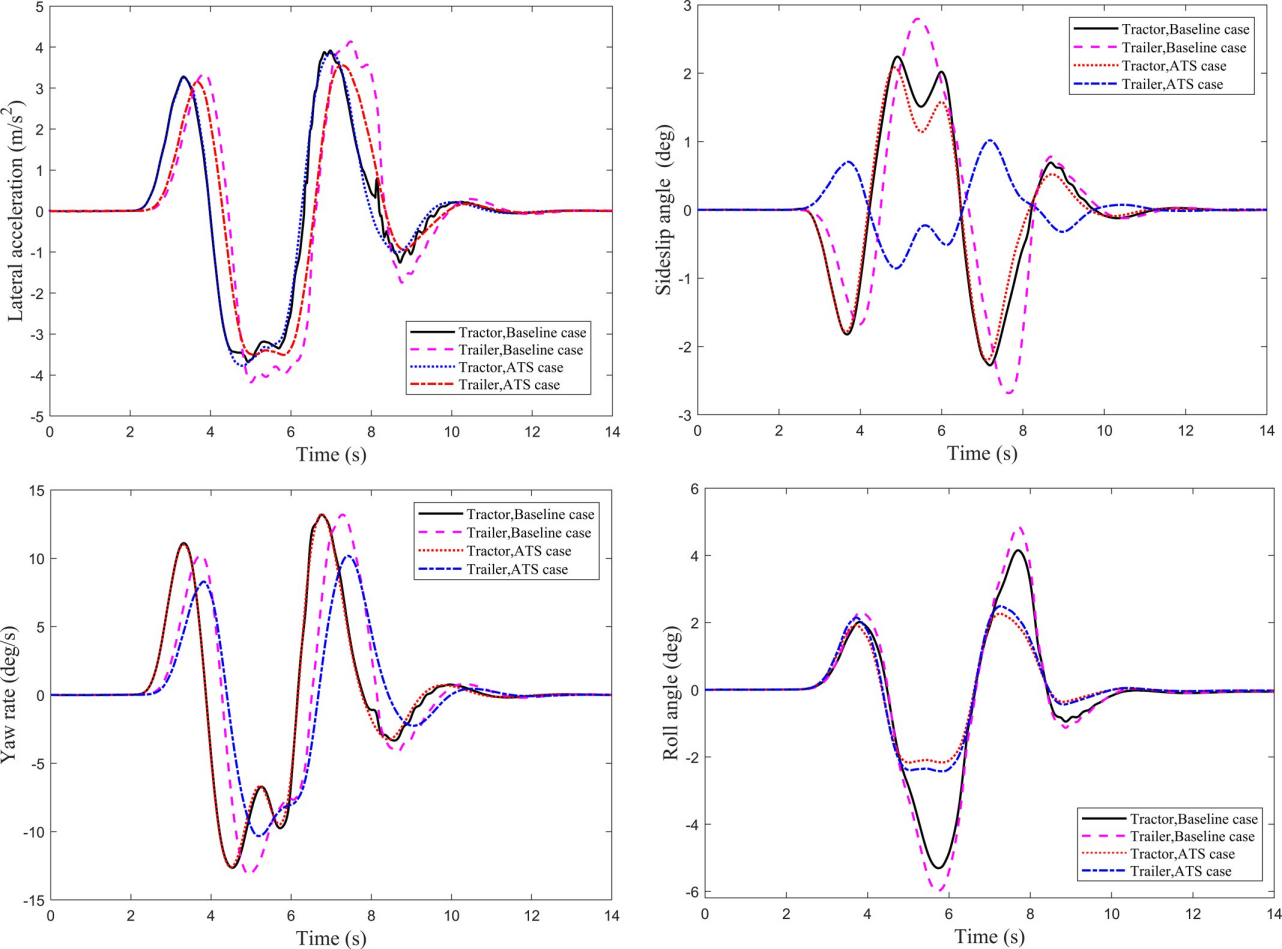
Dynamic responses of the tractor-semitrailer under the DLC maneuver at 80 km/h. (*a*) Lateral acceleration. (*b*) Sideslip angle. (*c*) Yaw rate. (*d*) Roll angle.

For tractor-semitrailers, the double lane-change (DLC) maneuver is another common driving condition at high speeds. In this subsection, the DLC maneuver of the tractor-semitrailer is performed at 80 km/h and 88 km/h, respectively. The simulation time step is set to 0.001s, and the simulation time is 14 s and 12 s, respectively. The trajectory tracking performance of the trailer and the lateral stability of the tractor-semitrailer with an ATS system are compared with the baseline case. Four dynamic responses of the tractor-semitrailer under the DLC maneuver at 80 km/h are shown in Fig 5. It can be seen that, except for the roll angle, the dynamic responses of the trailer all lag behind those of the tractor. The designed LQR controller has a significant impact on the sideslip angle and roll angle of the trailer. The ATS system reduces the maximum sideslip angle at the trailer’s CG from 2.79° to 1.01°, decreased by 63.8%, and reduces the maximum roll angle of the trailer from 5.98° to 2.42°, decreased by 59.5%. Moreover, the LQR controller reduces the lateral acceleration and yaw rate of the trailer to a certain extent. Compared with the baseline case, for the trailer with the ATS system, the peak values on the lateral acceleration curve and the yaw rate curve are decreased by 14.8% and 21.7%, respectively. In addition, no matter for the baseline or ATS case, the oscillation phenomenon appears in the dynamic response curves within a certain period. However, for the ATS case, the dynamic responses can reach a new steady state faster.

**Fig 6:**
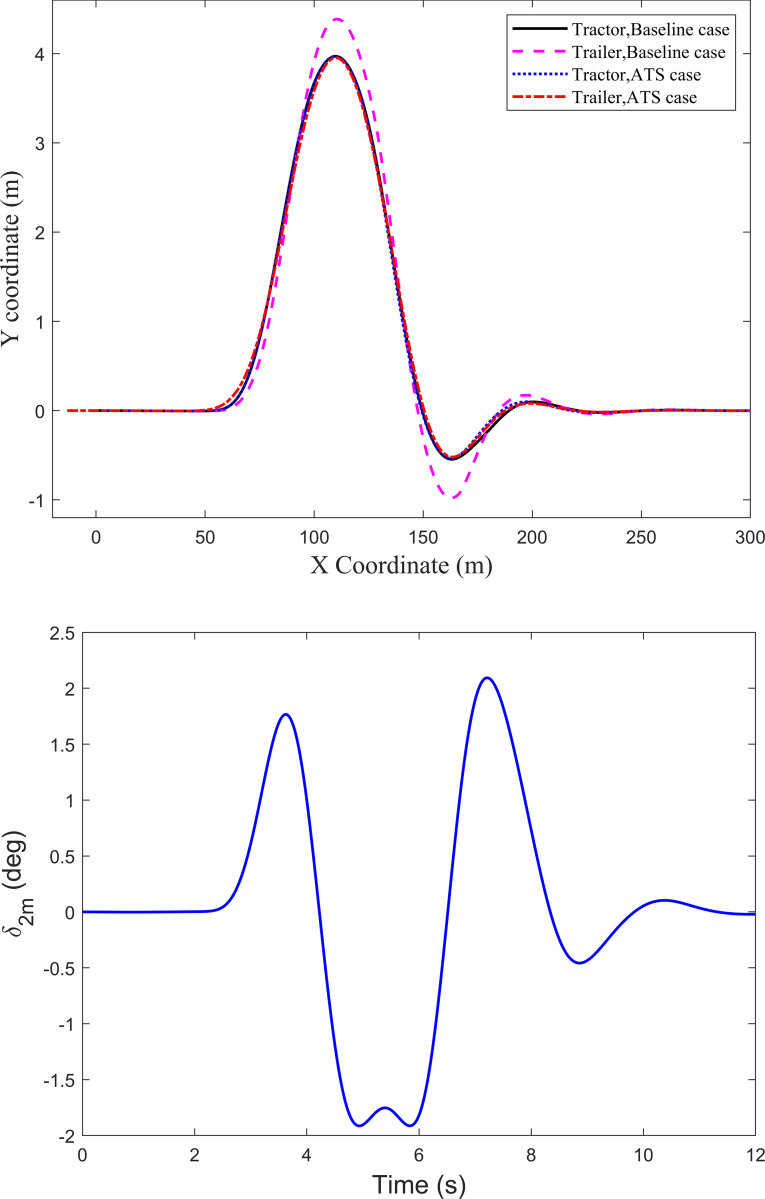
Axle trajectories and the active steering angle under the DLC maneuver at 80 km/h. (*a*) Axle trajectories. (*b*) Active steering angle *δ*_2m_.

When the tractor-semitrailer runs at 80 km/h under the DLC maneuver, trajectories of axles A1 and A6 are shown in Fig 6(A). For the baseline case, there is apparent trajectory deviation between axles A6 and A1, and the peak value of the trajectory deviation is 0.416 m. However, for the ATS case, axle A6 can track the trajectory of axle A1 very well, and the trajectory deviation between the two axles is almost zero. Obviously, the designed LQR controller not only improves the lateral stability of the tractor-semitrailer, but also dramatically increases the trajectory tracking performance of the trailer under the high-speed DLC maneuver. To understand the change of the middle-axle active steering angle *δ*_2m_ of the trailer under the DLC maneuver at 80km/h, Fig 6(B) shows its time history curve.

**Fig 7:**
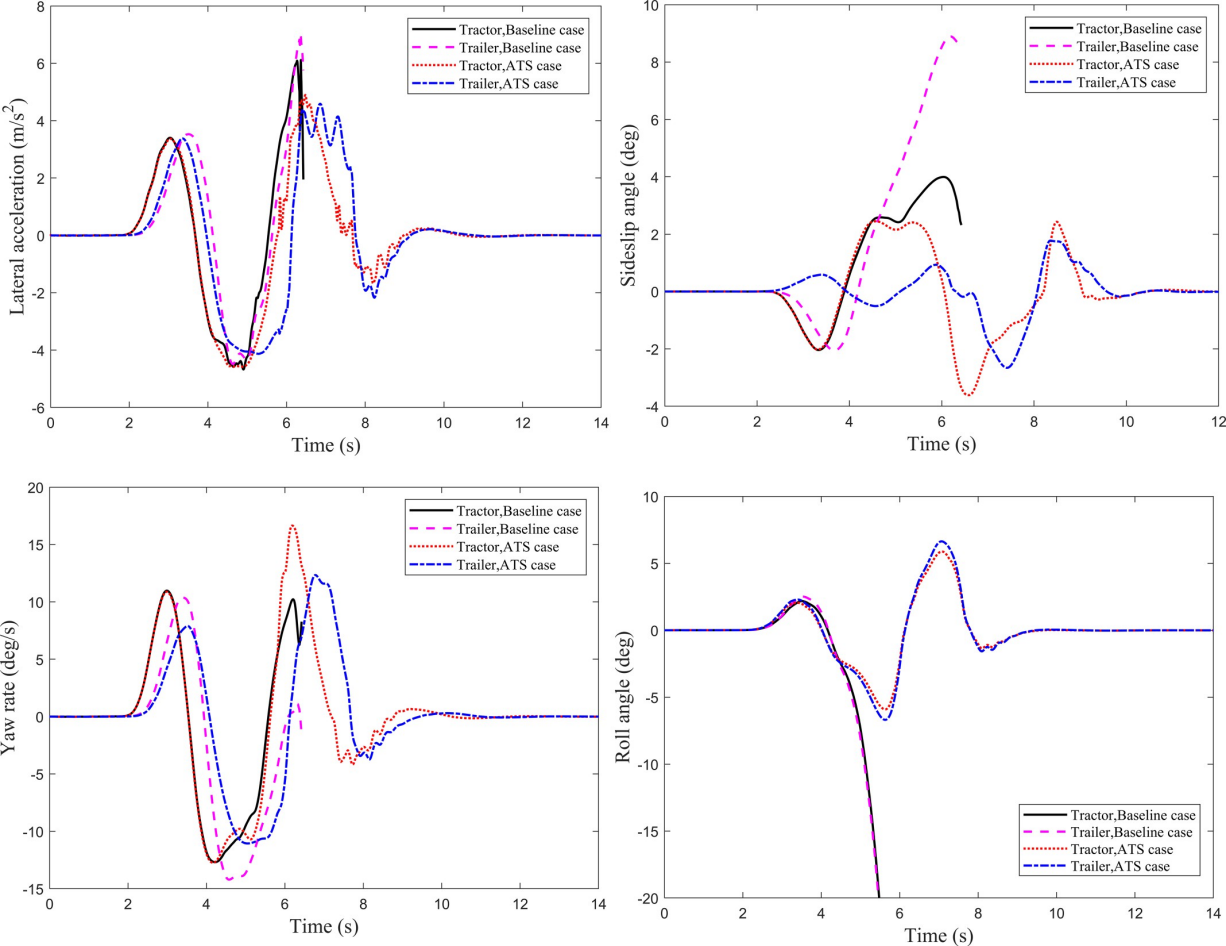
Dynamic responses of the tractor-semitrailer under the DLC maneuver at 88 km/h. (*a*) Lateral acceleration. (*b*) Sideslip angle. (*c*) Yaw rate. (*d*) Roll angle.

To further evaluate the effectiveness of the designed LQR controller, the DLC maneuver is performed at the speed at which the tractor-semitrailer without ATS will roll over. Fig 7 shows the time history of the dynamic responses of the tractor-semitrailer under the DLC maneuver at 88 km/h. It can be seen that for the baseline case, the dynamic response curves disappear from the figures after the simulation time of 6.4s, which means after that moment, the tractor-semitrailer without ATS has rolled over. However, for the tractor-semitrailer with ATS, although the dynamic response curves fluctuate to a certain extent during lane change, it can still reach a new steady state after the simulation time of 9 s.

**Fig 8:**
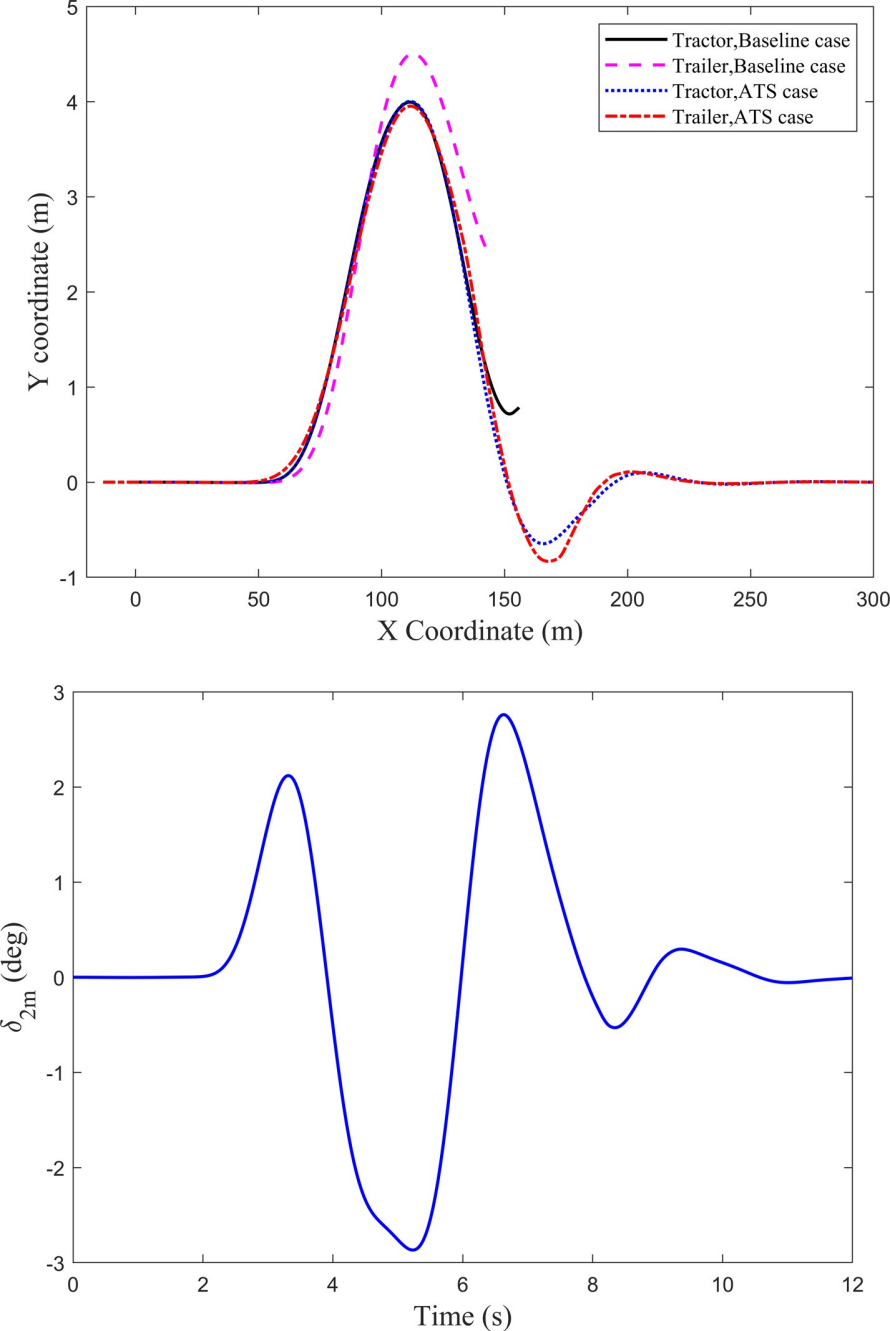
Axle trajectories and the active steering angle under the DLC maneuver at 88 km/h. (*a*) Axle trajectories. (*b*) Active steering angle *δ*_2m_.

Fig 8(A) shows trajectories of axles A1 and A6 when the tractor-semitrailer runs at 88 km/h under the DLC maneuver. For the baseline case, the trajectory curves of axles A1 and A6 disappear from the figure due to the tractor-semitrailer rollover, and the trailer rolls over before the tractor. The trailer rolls over when the longitudinal displacement of simulation is 142.6m, and the tractor rolls over when that is 155.8m. However, for the ATS case, except for a little trajectory deviation between axles A1 and A6 in a short time, axle A6 can still track the trajectory of axle A1 very well. To understand the change of the middle-axle active steering angle *δ*_2m_ of the trailer under the DLC maneuver at 88km/h, Fig 8(B) shows its time history curve.

Overall, under the high-speed DLC maneuver, the designed LQR controller can still make the tractor-semitrailer reach a new steady state in a short time. The designed LQR controller can improve the high-speed lateral stability of the tractor-semitrailer and the trajectory tracking performance of the trailer at the same time.

## 5 Conclusions

An ATS controller is designed to improve the lateral stability of the tractor-semitrailer and the trajectory tracking performance of the trailer. Based on the co-simulation platform, the effectiveness of the designed controller is examined under the SLC and DLC maneuvers. The main conclusions are drawn as follows.

1. The performance test results under the SLC maneuver show that the designed LQR controller has no apparent effect on the tractor trajectory and dynamic responses except the roll angle, and it can significantly improve the lateral stability and trajectory tracking performance of the trailer. Compared with the tractor-semitrailer without the ATS system, the tractor-semitrailer equipped with the ATS system has a reduction of 31.9%, 71.7%, 26.6%, and 40.8% in the peak values of the lateral acceleration, sideslip angle, yaw rate and roll angle of the trailer, respectively. The trajectory deviation between the rearmost axle A6 of the trailer and the front axle A1 of the tractor is reduced by 58.2%. Moreover, the designed LQR controller can suppress the shimmy phenomenon of the tractor-semitrailer in the process of SLC maneuver.
2. The performance test results under the DLC maneuver at 80 km/h show that the designed LQR controller has more significant impact on the trailer’s sideslip angle and roll angle than on the trailer’s lateral acceleration and yaw rate. Compared with the tractor-semitrailer without ATS, the tractor-semitrailer with ATS has a reduction of 63.8% and 59.5% in the peak values of the sideslip angle and roll angle, and 14.8% and 21.7% in the peak values of the lateral acceleration and yaw rate. The rearmost axle A6 of the trailer can track the trajectory of the front axle A1 of the tractor very well and the trajectory deviation between the two axles is almost zero.
3. Under the DLC maneuver at 88 km/h, the tractor-semitrailer without ATS rolls over during lane change, while under the same test condition, the tractor-semitrailer equipped with ATS can still reach a new steady state after a brief shimmy. The rearmost axle A6 of the trailer can track the trajectory of the front axle A1 of the tractor very well.

This article carried out the high-speed safety of the tractor-semitrailer mainly based on the numerical simulation method. The most direct natural extension involves carrying out physical experiments through hardware-in-the loop tests or field tests to further verify the analytical results and numerical simulation in this study. Possible future work also includes the development of more effective intelligent control algorithms for tractor-semitrailers, the cooperative control of the tractor and the trailer, and the chassis integrated control of the tractor-semitrailers.

## Supporting information

S1 Appendix Description and nominal values of some parameters for the tractor-semitrailer. (DOCX)

S2 Appendix Matrices definition and the non-zero elements of the matrices. (DOCX)
