# Supplementary material for: High-speed lateral stability and trajectory tracking performance for a tractor-semitrailer with active trailer steering
Source: PLoS One. 2022 Nov 14;17(11):e0277358. doi: 10.1371/journal.pone.0277358 (PMC9662746; doi:10.1371/journal.pone.0277358)
Supplement: S1 Appendix — (DOCX) [file pone.0277358.s001.docx]

**Appendix I：**Description and nominal values of some parameters for the tractor-semitrailer

| **Symbol** | **Description** | **Nominal value** |
| --- | --- | --- |
| *m*_1_ | Total mass of the tractor | 6360 kg |
| *m*_1s_ | Sprung mass of the tractor | 4455 kg |
| *m*_2_ | Total mass of the semitrailer | 25910 kg |
| *m*_2s_ | Sprung mass of the semitrailer | 23840 kg |
| *a*_1_ | Distance between the CG of the tractor and its front axle | 2.35 m |
| *b*_1_ | Distance between the CG of the tractor and its middle axle | 1.15 m |
| *c*_1_ | Distance between the hitch point and the middle axle of the tractor | 0.64 m |
| *d*_1_ | Distance between the hitch point and the rear axle of the tractor | 0.64 m |
| *a*_2_ | Distance between the hitch point and the CG of the semitrailer | 5.61 m |
| *b*_2_ | Distance between the CG of the semitrailer and its front axle | 1.11 m |
| *c*_2_ | Distance between the front axle and middle axle of the semitrailer | 1.20 m |
| *d*_2_ | Distance between the rear axle and middle axle of the semitrailer | 1.20 m |
| *h*_1_*_s_* | Height of the CG of the tractor sprung mass | 1.18 m |
| *h*_2_*_s_* | Height of the CG of the semitrailer sprung mass | 2.19 m |
| *h*_1_*_r_* | Height of the roll center of the tractor sprung mass | 0.61 m |
| *h*_2_*_r_* | Height of the roll center of the semitrailer sprung mass | 1.02 m |
| *h*_1_*_sr_* | Distance between the CG and the roll center of the tractor sprung mass | 0.57m |
| *h*_2_*_sr_* | Distance between the CG and the roll center of the semitrailer sprung mass | 1.17 m |
| *h_p_* | Height of the hitch point | 1.10 m |
| *I*_1_*_zz_* | Yaw moment of inertia of the tractor whole mass | 45075.9 kg m^2^ |
| *I*_1_*_sxx_* | Roll moment of inertia of the tractor sprung mass | 2283.9 kg m^2^ |
| *I*_1_*_sxz_* | Roll-yaw product of inertia of the tractor sprung mass | 1626 kg m^2^ |
| *I*_2_*_zz_* | Yaw moment of inertia of the semitrailer whole mass | 285516 kg m^2^ |
| *I*_2_*_sxx_* | Roll moment of inertia of the semitrailer sprung mass | 21802.3 kg m^2^ |
| *I*_2_*_sxz_* | Roll-yaw product of inertia of the semitrailer sprung mass | 0 kg m^2^ |
| *K*_1_^*^ | Roll stiffness of the tractor | 1631140 N m/rad |
| *K*_2_^*^ | Roll stiffness of the semitrailer | 4265880 N m/rad |
| *K*_12_ | Roll stiffness of the articulation joint between the tractor and semitrailer | 5729578 N m/rad |
| *C*_1_^*^ | Roll damping of the tractor’s suspension | 48150 N m s/rad |
| *C*_2_^*^ | Roll damping of the semitrailer’s suspension | 45000 N m s/rad |
| *k*_1_*_f_* | Tire cornering stiffness of the tractor front axle | -231430 N/rad |
| *k*_1_*_m_* | Tire cornering stiffness of the tractor middle axle | -520000 N/rad |
| *k*_1_*_r_* | Tire cornering stiffness of the tractor rear axle | -520000 N/rad |
| *k*_2_*_f_* | Tire cornering stiffness of the semitrailer front axle | -553000 N/rad |
| *k*_2_*_m_* | Tire cornering stiffness of the semitrailer middle axle | -553000 N/rad |
| *k*_2_*_r_* | Tire cornering stiffness of the semitrailer rear axle | -553000 N/rad |
