# Supplementary material for: High-speed lateral stability and trajectory tracking performance for a tractor-semitrailer with active trailer steering
Source: PLoS One. 2022 Nov 14;17(11):e0277358. doi: 10.1371/journal.pone.0277358 (PMC9662746; doi:10.1371/journal.pone.0277358)
Supplement: S2 Appendix — (DOCX) [file pone.0277358.s002.docx]

**Appendix II:** Matrices definition and the non-zero elements of the matrices

 (9)

In Eq (9), matrices ***A*** = ***M*^-1^*K***, ***B*** = ***M^-1^N***, and ***B*_0_*= M^-1^H.*** The matrices ***M***, ***K***, ***N***, ***H*** and the non-zero elements are given below.

$$\mathbf{M}=\left[\begin{matrix} m_{11} & m_{12} & 0 & m_{14} & 0 & 0 & 0 & 0 \\ m_{21} & m_{22} & m_{23} & m_{24} & 0 & 0 & 0 & 0 \\ 0 & 0 & 1 & 0 & 0 & 0 & 0 & 0 \\ 0 & 0 & 0 & 0 & m_{45} & m_{46} & 0 & m_{48} \\ 0 & 0 & 0 & 0 & m_{55} & m_{56} & m_{57} & m_{58} \\ 0 & 0 & 0 & 0 & 0 & 0 & 1 & 0 \\ m_{71} & 0 & 0 & m_{74} & m_{75} & 0 & 0 & m_{78} \\ 1 & m_{82} & 0 & m_{84} & -1 & m_{86} & 0 & m_{88} \end{matrix}\right]$$

In matrix ***M***, the non-zero elements are given as:

| $m_{11}=m_{1}v_{x1}(b_{1}+c_{1})$, | $m_{12}=I_{1zz}$, |
| --- | --- |
| $m_{14}=-[m_{1s}h_{1sr}(b_{1}+c_{1})+I_{1sxz}]$, | $m_{21}=m_{1}v_{x1}h_{1cr}-m_{1s}h_{1sr}v_{x1}$, |
| $m_{22}=-I_{1sxz}$, | $m_{23}=C_{1}^{\text{*}}$, |
| $m_{24}=-m_{1s}h_{1sr}h_{1cr}\text{+}I_{1sxx}-2m_{1s}h_{1sr}^{2}$, | $m_{45}=m_{2}v_{x2}a_{2}$, |
| $m_{46}=-I_{2zz}$, | $m_{48}=-m_{2s}h_{2sr}a_{2}+I_{2sxz}$, |
| $m_{55}=m_{2}v_{x2}h_{2cr}-m_{2s}h_{2sr}v_{x2}$, | $m_{56}=-I_{2sxz}$, |
| $m_{57}=C_{2}^{\text{*}}$, | $m_{58}=-m_{2s}h_{2sr}h_{2cr}+I_{2sxx}+2m_{2s}h_{2sr}^{2}$, |
| $m_{71}=m_{1}v_{x1}$, | $m_{74}=-m_{1s}h_{1sr}$, |
| $m_{75}=m_{2}v_{x2}$, | $m_{78}=-m_{2s}h_{2sr}$, |
| $m_{82}=-\left(b_{1}+c_{1}\right)/v_{x1}$, | $m_{84}=-h_{1cr}/v_{x1}$, |
| $m_{86}=-a_{2}/v_{x2}$, | $m_{88}=-h_{2cr}/v_{x2}$. |

$$\mathbf{K}=\left[\begin{matrix} a_{11} & a_{12} & 0 & 0 & 0 & 0 & 0 & 0 \\ a_{21} & a_{22} & a_{23} & 0 & 0 & 0 & a_{27} & 0 \\ 0 & 0 & 0 & 1 & 0 & 0 & 0 & 0 \\ 0 & 0 & 0 & 0 & a_{45} & a_{46}+a'_{46} & 0 & 0 \\ 0 & 0 & a_{53} & 0 & a_{55} & a_{56}+a'_{56} & a_{57} & 0 \\ 0 & 0 & 0 & 0 & 0 & 0 & 0 & 1 \\ a_{71} & a_{72} & 0 & 0 & a_{75} & a_{76}+a'_{76} & 0 & 0 \\ 0 & \text{-}1 & 0 & 0 & 0 & 1 & 0 & 0 \end{matrix}\right]$$

In matrix ***K***, the non-zero elements are given as:

| $a_{11}=(a_{1}+b_{1}+c_{1})k_{1f}+c_{1}k_{1m}-d_{1}k_{1r}$, | $a_{21}=h_{1cr}(k_{1f}+k_{1m}+k_{1r})$, |
| --- | --- |
| $a_{12}=[a_{1}(a_{1}+b_{1}+c_{1})k_{1f}-b_{1}c_{1}k_{1m}+d_{1}(b_{1}+c_{1}+d_{1})k_{r}]/v_{x1}-m_{1}v_{1}(b_{1}+c_{1})$, | |
| $a_{22}=h_{1cr}[a_{1}k_{1f}-b_{1}k_{1m}-(b_{1}+c_{1}+d_{1})k_{1r}]/v_{x1}-m_{1}v_{1}h_{1cr}+m_{1s}v_{1}h_{1sr}$, | |
| $a_{23}=m_{1s}gh_{1sr}-K_{1}^{*}-K_{12}$, | $a_{27}=K_{12}$, |
| $a_{45}=(a_{2}+b_{2})k_{2f}+(a_{2}+b_{2}+c_{2})k_{2m}+(a_{2}+b_{2}+c_{2}+d_{2})k_{2r}$, | |
| $a_{46}=[-b_{2}(a_{2}+b_{2})k_{2f}-(b_{2}+c_{2})(a_{2}+b_{2}+c_{2})k_{2m}-(b_{2}+c_{2}+d_{2})(a_{2}+b_{2}+c_{2}+d_{2})k_{2r}]/v_{x2}-m_{2}v_{2}a_{2}$, | |
| $a'_{46}=-(a_{2}+b_{2})c_{2}k_{2f}/v_{x2}+(a_{2}+b_{2}+c_{2}+d_{2})d_{2}k_{2r}/v_{x2}$, | |
| $a_{53}=K_{12}$, | $a_{55}=h_{2cr}(k_{2f}+k_{2m}+k_{2r})$, |
| $a_{56}=h_{2cr}[-b_{2}k_{2f}-(b_{2}+c_{2})k_{2m}-(b_{2}+c_{2}+d_{2})k_{2r})]/v_{x2}-m_{2}v_{2}h_{2cr}+m_{2s}v_{2}h_{2sr}$, | |
| $a'_{56}=-h_{2cr}c_{2}k_{2f}/v_{x2}+h_{2cr}d_{2}k_{2r}/v_{x2}$, | |
| $a_{57}=m_{2s}gh_{2sr}-K_{1}^{*}-K_{12}$, | $a_{71}=k_{1f}+k_{1m}+k_{1r}$, |
| $a_{72}=\left[a_{1}k_{1f}-b_{1}k_{1m}-(b_{1}+c_{1}+d_{1})k_{1r}\right]/v_{x1}-m_{1}v_{x1}$, | $a_{75}=k_{2f}+k_{2m}+k_{2r}$, |
| $a_{76}=\left[-b_{2}k_{2f}-(b_{2}+c_{2})k_{2m}-(b_{2}+c_{2}+d_{2})k_{2r})\right]/v_{x2}-m_{2}v_{x2}$, | |
| $a'_{76}=-c_{2}k_{2f}/v_{x2}+d_{2}k_{2r}/v_{x2}$. | |

$$\mathbf{N}=\left[\begin{matrix} 0 & 0 & 0 & b'_{4} & b'_{5} & 0 & b'_{7} & 0 \end{matrix}\right]^{\text{T}}$$

In matrix ***N***, the non-zero elements are given as:

| $b'_{4}\text{=}-(a_{2}+b_{2})k_{2f}-(a_{2}+b_{2}+c_{2})k_{2m}-(a_{2}+b_{2}+c_{2}+d_{2})k_{2r}$, | |
| --- | --- |
| $b'_{5}\text{=}-h_{2cr}(k_{2f}+k_{2m}+k_{2r})$, | $b'_{7}\text{=}-(k_{2f}+k_{2m}+k_{2r})$. |

$$\mathbf{H}=\left[\begin{matrix} b_{11} & 0 & 0 & 0 \\ b_{21} & 0 & 0 & 0 \\ 0 & 0 & 0 & 0 \\ 0 & b_{42} & b_{43} & b_{44} \\ 0 & b_{52} & b_{53} & b_{54} \\ 0 & 0 & 0 & 0 \\ b_{71} & b_{72} & b_{73} & b_{74} \\ 0 & 0 & 0 & 0 \end{matrix}\right]$$

In matrix ***H***, the non-zero elements are given as:

| $b_{11}=-(a_{1}+b_{1}+c_{1})k_{1f}$, | $b_{21}=-h_{1cr}k_{1f}$, |
| --- | --- |
| $b_{42}=-(a_{2}+b_{2})k_{2f}$, | $b_{43}=-(a_{2}+b_{2}+c_{2})k_{2m}$, |
| $b_{44}=-(a_{2}+b_{2}+c_{2}+d_{2})k_{2r}$, | $b_{52}=-h_{2cr}k_{2f}$, |
| $b_{53}=-h_{2cr}k_{2m}$, | $b_{54}=-h_{2cr}k_{2r}$, |
| $b_{71}=-k_{1f}$, | $b_{72}=-k_{2f}$, |
| $b_{73}=-k_{2m}$, | $b_{74}=-k_{2r}$. |
